# The impact of body condition after calving on metabolism and milk progesterone profiles in two breeds of dairy cows

**DOI:** 10.1186/s13028-016-0251-2

**Published:** 2016-10-20

**Authors:** Lisa A. O’Hara, Renée Båge, Kjell Holtenius

**Affiliations:** 1 Department of Animal Nutrition and Management, Swedish University of Agricultural Sciences, PO Box 7045, 750 07 Uppsala, Sweden; 2 Department of Clinical Sciences, Swedish University of Agricultural Sciences, PO Box 7054, 750 07 Uppsala, Sweden

**Keywords:** Dairy breed, Body condition, Progesterone

## Abstract

**Background:**

Optimal body condition in early lactation is generally accepted as a prerequisite for good reproductive performance. Examination of milk progesterone profiles offers an objective method for characterization of postpartum ovarian activity in dairy cows. The present study investigated the relationship between body condition after calving, some metabolic parameters in blood plasma, and fertility, as reflected by milk progesterone profiles in the two dairy breeds Swedish Red (SR) and Swedish Holstein (SH).

**Results:**

Multiparous dairy cows (n = 73) of SR and SH breeds were selected and divided into three groups based on their body condition score (BCS) after parturition. Selected plasma metabolites were determined, milk progesterone profiles were identified and body condition was scored. Over-conditioned cows and atypical progesterone profiles were more common among SR cows. Insulin sensitivity was lower and IGF 1 higher among SR cows. Insulin was positively related to body condition, but not related to breed.

**Conclusions:**

Atypical progesterone profiles were more common and insulin sensitivity lower in SR than in SH cows, but the SR breed had a higher proportion of over-conditioned SR cows. It is reasonable to assume that breed differences in body condition contributed to these results.

## Findings

The optimum early lactation body condition score (BCS) for successful return to estrus appears to be between 3.0 and 3.5 as reviewed by Roche et al. [1].

Examination of milk progesterone profiles offers an objective method for characterization of postpartum ovarian activity in dairy cows [2]. Previous studies have shown that cows of the Swedish Holstein (SH) breed have a higher risk of atypical progesterone profiles than Swedish Red (SR) cows [2].

The aim of the present study was to investigate the relationship between body condition at calving, some metabolic parameters in blood plasma and fertility, as reflected by milk progesterone profiles in two dairy breeds (SR and SH).

Multiparous SH (n = 33) and SR (n = 40) dairy cows calving were selected from the Swedish Livestock Research Centre, Lövsta, and housed indoors in a loose-house system. Cows that got sick during the experiment were excluded if they had to be kept in individual boxes for more than a week. They were milked twice daily in an automatic rotary system (DeLaval AMR™, Tumba, Sweden). Silage was provided ad libitum, while the supply of concentrate was restricted to 3 kg immediately post-partum and then increased with 0.5 kg/day to 13.5 kg/d. Only apparently clinically healthy cows yielding more than 15 kg milk at 10 weeks prior to expected calving date, and not treated with antibiotics at drying off, were included. Body condition was scored twice, at 1 and 4 weeks after parturition, using a 5-point scale with 0.25-point increments, where 1 indicates emaciation and 5 indicates obesity [3]. The cows were divided into three groups based on their BCS in the first week after parturition; thin (<3.25), adequate (3.25–3.75) and over-conditioned cows (>3.75).

Blood was sampled in the second week after parturition from a coccygeal artery or vein into heparinized 10 ml vacuum tubes with lithium heparin as anticoagulant (Venoject, Terumo Europe N.V., Leuven, Belgium). The samples were centrifuged at +4 °C within 1 h of sampling and the plasma samples were stored at −20 °C until further analysis. After thawing, plasma samples were analysed for glucose using enzymatic colorimetric tests (Glucose liquicolor, Human, Wiesbaden, Germany) and spectrophotometry (Ultrospec K, Boule Nordic, Huddinge, Sweden). The insulin concentration was analysed using a commercial enzyme immunoassay method adapted for bovines (Mercodia Ultrasensitive Bovine Insulin ELISA, Mercodia AB, Uppsala, Sweden) and the concentration of non-esterified fatty acids (NEFA) using an enzymatic colorimetric test (NEFA C, Wako Chemicals GmbH, Neuss, Germany). The Revised Quantitative Insulin Sensitivity Check Index (RQUICKI) was used to estimate insulin sensitivity as described by Holtenius and Holtenius [4]. IGF-1 was analyzed using a commercial enzyme immunoassay (Bovine Insulin-like Growth Factor 1 ELISA Cat. No. KT-18278 Kamaya Medical Company Seattle, WA, USA).

Progesterone concentration was determined in milk samples collected twice weekly from 2 to 12 weeks postpartum by a commercial ELISA (Ridgeway Science Ltd., Alvington, UK). The profiles were classified according to Petersson et al. [2] as normal or atypical (delayed, interrupted or prolonged luteal activity), with the limit for luteal activity set at a milk progesterone concentration >3 ng/ml.

The software Minitab 17.1.0 was used for statistical analyses. Body condition was compared against breed (SR and SH) and progesterone profiles (normal and atypical) using Chi square tests. Breed and progesterone profiles were compared against the continuous variables by binary logistic regression, while body condition class was compared against the continuous variables using ordinal logistic regression. Differences were defined as significant at P < 0.05.

**Fig. 1:**
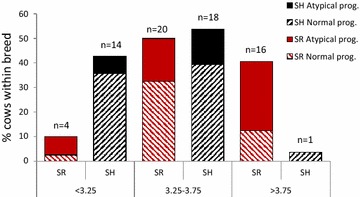
Progesterone profiles in Swedish Red Breed, (n = 40) and Swedish Holstein Breed, (n = 33) cows of different body condition classes (week 4)

The proportion of thin, adequate and over-conditioned cows differed between breeds (P < 0.001), with 43, 54 and 4 %, respectively, among SH cows and 11, 53 and 37 %, respectively, among SR cows. The proportion of cows with atypical progesterone profiles was higher among SR cows than among SH cows (45.6 vs. 19.7 %; P < 0.001). The proportion of cows with atypical progesterone profiles related to body condition class in week 4 is shown in Fig. 1. The number of days from calving to onset of luteal activity was neither affected by breed or by body condition class. Six cows, five SR and one SH, showed consistently low progesterone levels throughout the 12 weeks sampling period and they were classified cows with atypical profiles.

The influence of breed on body condition and metabolism-related parameters is shown in Table 1. Cows of the SR breed had higher BCS at 1 and 4 weeks after calving than SH cows, but the difference in BCS between these two measurement occasions was not affected by breed. The body condition was scored according to a chart for dual purpose cattle [3], and it is possible that the body condition of the Holstein cows were underestimated. RQUICKI was lower and IGF 1 higher among SR cows when compared with SH cows. None of the other metabolic parameters determined was related to breed.

**Table 1:** Influence of breed on body condition score (BSC) and selected plasma parameters in cows in their second week of lactation

	Swedish Red		Swedish Holstein		P value
	Mean	SEM	Mean	SEM	
BCS 1	3.69	0.06	3.22	0.08	<0.001
BCS 4	3.57	0.08	3.04	0.07	<0.001
BC difference*	−0.14	0.08	−0.08	0.07	0.61
NEFA µeq/ml	0.30	0.05	0.38	0.02	0.07
Insulin ng/ml	0.38	0.04	0.30	0.06	0.28
Glucose mM	3.19	0.08	3.10	0.08	0.41
IGF 1 ng/ml	80	3.6	65	4.6	0.009
RQUICKI	0.49	0.01	0.55	0.03	0.02

* Difference in body condition between week 1 (BCS 1) and week 4 (BCS 4) after parturition

*SEM* standard error of mean

The difference in body condition between 1 and 4 weeks after parturition and the various metabolic factors determined were not related to type of progesterone profile (Table 2). Body condition loss between 1 and 4 weeks after calving tended to be related to initial body condition. The group of over-conditioned cows tended to lose more body condition than thin cows and cows in adequate condition (Table 3). Insulin, IGF1 and RQUICKI were all significantly related to body condition group with higher insulin and IGF concentrations and lower RQUICKI value in over-conditioned cows.

**Table 2:** Selected plasma parameters and difference in BCS in cows with normal and atypical milk progesterone profiles

	Normal progesterone profile		Atypical progesterone profile		P value
	Mean	SEM	Mean	SEM	
BCS difference*	−0.19	0.08	−0.0	0.08	0.29
NEFA µeq/ml	0.29	0.05	0.35	0.02	0.23
Insulin ng/ml	0.33	0.05	0.36	0.05	0.68
Glucose mM	3.11	0.09	3.20	0.07	0.51
IGF 1 ng/ml	77	3.5	71	4.4	0.24
RQUICKI	0.51	0.02	0.51	0.2	0.98

* Difference in body condition score between weeks one and four after parturition

*SEM* standard error of mean

**Table 3:** Selected plasma parameters and differences in BCS in cows in different body condition score classes (<3.25 (thin); 3.25–3.75 (adequate); >3.75 (over-conditioned)

	<3.25		3.25–3.75		>3.75		P value
	Mean	SEM	Mean	SEM	Mean	SEM	
BCS difference*	−0.02	0.07	−0.08	0.07	−0.36	0.13	0.086
NEFA µeq/ml	0.30	0.031	0.38	0.02	0.47	0.038	0.55
Insulin ng/ml	0.24	0.07	0.36	0.06	0.50	0.08	0.035
Glucose mM	3.12	0.15	3.25	0.07	3.00	0.14	0.51
IGF 1 ng/ml	63	6.9	74	4.8	86	4.4	0.013
RQUICKI	0.56	0.03	0.49	0.02	0.48	0.02	0.020

* Difference in body condition score between weeks 1 and 4 after parturition

*SEM* standard error of mean

Overall, SR cows had a higher risk of having an atypical progesterone profile than SH cows. The fact that SR cows generally had higher body condition might have contributed to this result which was unexpected since previous studies have shown that SH cows have a higher risk of atypical progesterone profiles than SR cows [2]. One of the causes of delayed resumption of ovarian function in high-yielding dairy cows is a negative energy balance (NEB) during the postpartum period [5–7]. In the present study there was no difference in body condition loss between cows with different progesterone profiles. However, the average loss in BCS from week 1 to 4 after calving was low, which indicates that the NEB was limited. Cows classified as over-conditioned tended to lose more body condition than those classified as thin or in adequate condition, confirming previous observations that over-conditioned cows experience deeper postparturient NEB than thin and moderately conditioned cows [1, 8].

In general, fertility parameters have favourable associations with circulating concentrations of glucose, insulin and IGF-1 and unfavourable associations with NEFA, compounds that are all related to energy balance [9]. In the present study, none of these compounds was related to normal cyclicity, although SH cows had lower IGF 1 levels than SR cows and it is reported that reduced IGF 1 around calving can adversely influence fertility [10]. The lack of effect of the lower IGF 1 concentration on fertility in the SH cows is probably because their circulating concentrations were still high, so reduced concentrations never occurred [10]. Insulin sensitivity is also suggested to be related to ovarian function in dairy cows [11], but in the present study RQUICKI, a proxy for insulin sensitivity [4], was not related to ovarian function as determined by progesterone profiles. RQUICKI was highest in thin cows, in agreement with results of other studies [4, 12], suggesting that insulin sensitivity is related to body condition in lactating cows, as it is in heifers [13]. Cows of SH breed had higher RQUICKI values than SR cows, indicating higher insulin sensitivity. However, the SH cows were also thinner and the apparent higher insulin sensitivity among SH cows might not necessarily be a breed effect, but could be related to body condition.

In conclusion, atypical progesterone profiles were more common and insulin sensitivity lower in SR than in SH cows. However, the proportion of over-conditioned individuals was higher for SR cows and thus it was not possible to distinguish whether the differences in progesterone profile and insulin sensitivity were related to body condition, breed or both.

## Abbreviations

BCS: body condition score
IGF 1: insulin-like growth factor 1
NEFA: non-esterified fatty acids
RQUICKI: revised quantitative insulin sensitivity check index
SH: Swedish Holstein Breed
SR: Swedish Red Breed

## References

[1] Roche JR, Friggens NC, Kay JK, Fisher MW, Stafford KJ, Berry DP (2009). Invited review: body condition score and its association with dairy cow productivity, health, and welfare. J Dairy Sci.

[2] Petersson KJ, Gustafsson H, Strandberg E, Berglund B (2006). Atypical progesterone profiles and fertility in Swedish dairy cows. J Dairy Sci.

[3] Gillund P (1998). Holdvurdering av mjølkekyr—et nyttig verktøy i forebyggende helsearbeid.

[4] Holtenius P, Holtenius K (2007). A model to estimate insulin sensitivity in dairy cows. Acta Vet Scand.

[5] Lucy MC (2001). Reproductive loss in high-producing dairy cattle: where will it end?. J Dairy Sci.

[6] Beam SW, Butler WR (1999). Effects of energy balance on follicular development and first ovulation in postpartum dairy cows. J Reprod Fertil Suppl.

[7] Samarütel J, Waldmann A, Ling K, Jaakson H, Kaart T, Leesmäe A, Kärt O (2008). Relationships between luteal activity, fertility, blood metabolites and body condition score in multiparous Estonian Holstein dairy cows under different management. J Dairy Res.

[8] Holtenius K, Agenäs S, Delavaud C, Chilliard Y (2003). Effects of feeding intensity during the dry period. 2. Metabolic and hormonal responses. J Dairy Sci.

[9] Butler ST (2014). Nutritional management to optimize fertility of dairy cows in pasture-based systems. Animal.

[10] Wathes DC, Bourne N, Cheng Z, Mann GE, Taylor VJ, Coffey MP (2007). Multiple correlation analyses of metabolic and endocrine profiles with fertility in primiparous and multiparous cows. J Dairy Sci.

[11] Opsomer G, Wensing T, Laevens H, Coryn M, de Kruif A (1999). Insulin resistance: the link between metabolic disorders and cystic ovarian disease in high yielding dairy cows?. Anim Reprod Sci.

[12] Van Saun, R: Insulin sensitivity measures in lactating dairy cows and relationship to body condition score (Abstr 113). 26th World Buiatrics Congress. Santiago, Chile. 2010 (CD proceedings).

[13] McCann JP, Reimers TJ (1986). Effects of obesity on insulin and glucose metabolism in cyclic heifers. J Anim Sci.

